# Controlling metal–insulator transitions in reactively sputtered vanadium sesquioxide thin films through structure and stoichiometry

**DOI:** 10.1038/s41598-021-85397-x

**Published:** 2021-03-18

**Authors:** Einar B. Thorsteinsson, Seyedmohammad Shayestehaminzadeh, Arni S. Ingason, Fridrik Magnus, Unnar B. Arnalds

**Affiliations:** 1 Science Institute, University of Iceland, Dunhaga 3, 107, Reykjavik, Iceland; 2 Technovation Centre, AGC Glass Europe, Rue Louis Blériot 12, BE 6041 Gosselies, Belgium; 3 Grein Research ehf., Dunhaga 5, 107, Reykjavik, Iceland

**Keywords:** Surfaces, interfaces and thin films, Applied physics

## Abstract

We present a study of $\hbox {V}_{2}\hbox {O}_{3}$ thin films grown on *c*-plane $\hbox {Al}_{2}\hbox {O}_{3}$ substrates by reactive dc-magnetron sputtering. Our results reveal three distinct types of films displaying different metal–insulator transitions dependent on the growth conditions. We observe a clear temperature window, spanning 200 $^{\circ }$C, where highly epitaxial films of $\hbox {V}_{2}\hbox {O}_{3}$ can be obtained wherein the transition can be tuned by controlling the amount of interstitial oxygen in the films through the deposition conditions. Although small structural variations are observed within this window, large differences are observed in the electrical properties of the films with strong differences in the magnitude and temperature of the metal–insulator transition which we attribute to small changes in the stoichiometry and local strain in the films. Altering the sputtering power we are able to tune the characteristics of the metal–insulator transition suppressing and shifting the transition to lower temperatures as the power is reduced. Combined results for all the films fabricated for the study show a preferential increase in the *a* lattice parameter and reduction in the *c* lattice parameter with reduced deposition temperature with the film deviating from a constant volume unit cell to a higher volume.

## Introduction

Vanadium sesquioxide ($\hbox {V}_{2}\hbox {O}_{3}$) is a transition metal oxide which, like several other such oxides, exhibits a structural phase transition with temperature^1^. In bulk form $\hbox {V}_{2}\hbox {O}_{3}$ undergoes the transition at around 155 K where its crystallographic structure changes from a rhombohedral phase at high temperatures to a low temperature monoclinic phase. Coupled to the structural phase transition is a change in the resistivity of the material from a metallic state to an insulating state at low temperature^2^, as well as a change in the magnetic state from a paramagnetic to an antiferromagnetic state. $\hbox {V}_{2}\hbox {O}_{3}$ is also a thermochromic material changing its optical properties during the transition^3,4^.

As opposed to bulk $\hbox {V}_{2}\hbox {O}_{3}$, which shows a sharp change in both structure and resistivity at the transition, thin films display transitions affected by the choice of substrate, fabrication method, deposition conditions and thickness^5–9^. Through these choices the scale and magnitude as well as the transition temperature can be controlled via strain in the film^10^ induced by the lattice mismatch between $\hbox {V}_{2}\hbox {O}_{3}$ and substrate material and through stoichiometry as the transition is sensitive to the amount of vanadium and oxygen deficiencies present in the film^11–14^. The morphology of the films also plays a large role as a nanotextured phase coexistence^15,16^ has been observed for $\hbox {V}_{2}\hbox {O}_{3}$ in thin film form, both using direct imaging^17^ as well as through secondary effects such as the modification of the coercivity of overlying magnetic layers^18^. The nanoscale structure has furthermore been observed utilizing nanoscopic contacts to investigate the resistivity of $\hbox {V}_{2}\hbox {O}_{3}$ films. These results show the metal to insulator transition to occur through avalanches as opposed to a smooth transition with the size of the observed jumps in resistivity following a power law behaviour^19^.

In this article we investigate how the structural and electrical properties of $\hbox {V}_{2}\hbox {O}_{3}$ thin films grown by reactive dc-magnetron sputtering can be controlled by the fabrication conditions. In order to achieve this we perform a systematic study of the effects of (1) substrate temperature, (2) $\hbox {O}_2$ flow into the chamber and (3) magnetron sputtering power on the overall film properties. We are able to identify how their properties can be tuned and controlled through these parameters and investigate the underlying crystallographic differences in the films and how they affect their properties. We observe a clear correlation between the structural properties and the controllable deposition parameters enabling tuning of the structural as well as electrical properties of the films. We show that the growth temperature is an important factor for the crystalline properties of the fabricated films and how it affects the MIT of the films strongly. Films grown at different temperatures display distinct MIT’s which can be classified into three types of transitions ranging from films showing a large hysteresis to films with a suppressed transition. Within an intermediate deposition temperature range (400–600 $^{\circ }\hbox {C}$), classified as type II, we observe a controllable transition wherein the amount of interstitial oxygen in the films can be used to tune the transition. Oxygen is known to occupy interstitial sites in transition metal oxides and results have shown that it affects the metal–insulator transition even for minute quantities^2,20^. Within bixbyite, a metastable polymorph of $\hbox {V}_{2}\hbox {O}_{3}$, oxygen has been confirmed to occupy interstitial sites with minimal changes in the structure^21^. Films displaying a transition of type II are highly sensitive to the oxygen stoichiometry with an increase in oxygen interstitials suppressing and shifting the transition to a lower temperature^11,20^. In this type, changes in the crystal structure of the film are limited but large differences can be obtained in the transition behaviour. Our results show that different growth parameters affect the films properties in a coupled manner with films grown at higher sputtering power and $\hbox {O}_2$ flow settings showing similar structural and electrical properties as films grown at lower sputtering power and $\hbox {O}_2$ flow. The results therefore reveal the possibility for detailed tuning of the structural properties, stoichiometry and metal–insulator transition via the deposition conditions.

## Results

### Growth temperature dependence

The primary factor in the crystalline properties of the $\hbox {V}_{2}\hbox {O}_{3}$ films is the substrate temperature during growth. For this study an initial characterization of several films grown at varying substrate temperatures was therefore performed and has been described elsewhere^11^. For $\hbox {V}_{2}\hbox {O}_{3}$ these studies have already revealed a temperature window where highly epitaxial films are obtained between roughly 400 $^{\circ }$C and 600 $^{\circ }$C. This temperature window is at substantially lower values than those used for fabricating epitaxial $\hbox {V}_{2}\hbox {O}_{3}$ thin films using other methods such as rf sputtering of compound targets and molecular beam epitaxy which are generally around 700 $^{\circ }$C^6,12,18,22–25^. Reactive dc magnetron sputtering is therefore a highly viable method for the fabrication of highly crystalline $\hbox {V}_{2}\hbox {O}_{3}$ films for both research and applications without the need for post-deposition annealing^26–28^.

All the films were grown at 0.4 Pa pressure with a 20 sccm flow rate for argon. Two different oxygen flow rates were used for this series, 1.4 sccm and 1.6 sccm. The sputtering power was kept at a constant 150 W and the temperature was varied from 350 $^{\circ }$C up to 670 $^{\circ }$C in 45 $^{\circ }$C steps. Although the fabrication conditions are varied substantially during growth, with respect to the chamber oxygen environment, substrate temperature and sputtering power, the main phase of the films was in all cases observed to be $\hbox {V}_{2}\hbox {O}_{3}$. All films reported in this article revealed a clear peak in X-ray diffraction scans corresponding to the $\hbox {V}_{2}\hbox {O}_{3}$[0006] lattice spacing. It should be noted that as the temperature is changing the reaction rate of the vanadium with the oxygen is also changing and, therefore, the stoichiometry is affected as well.

#### Reciprocal space mapping

**Figure 1:**
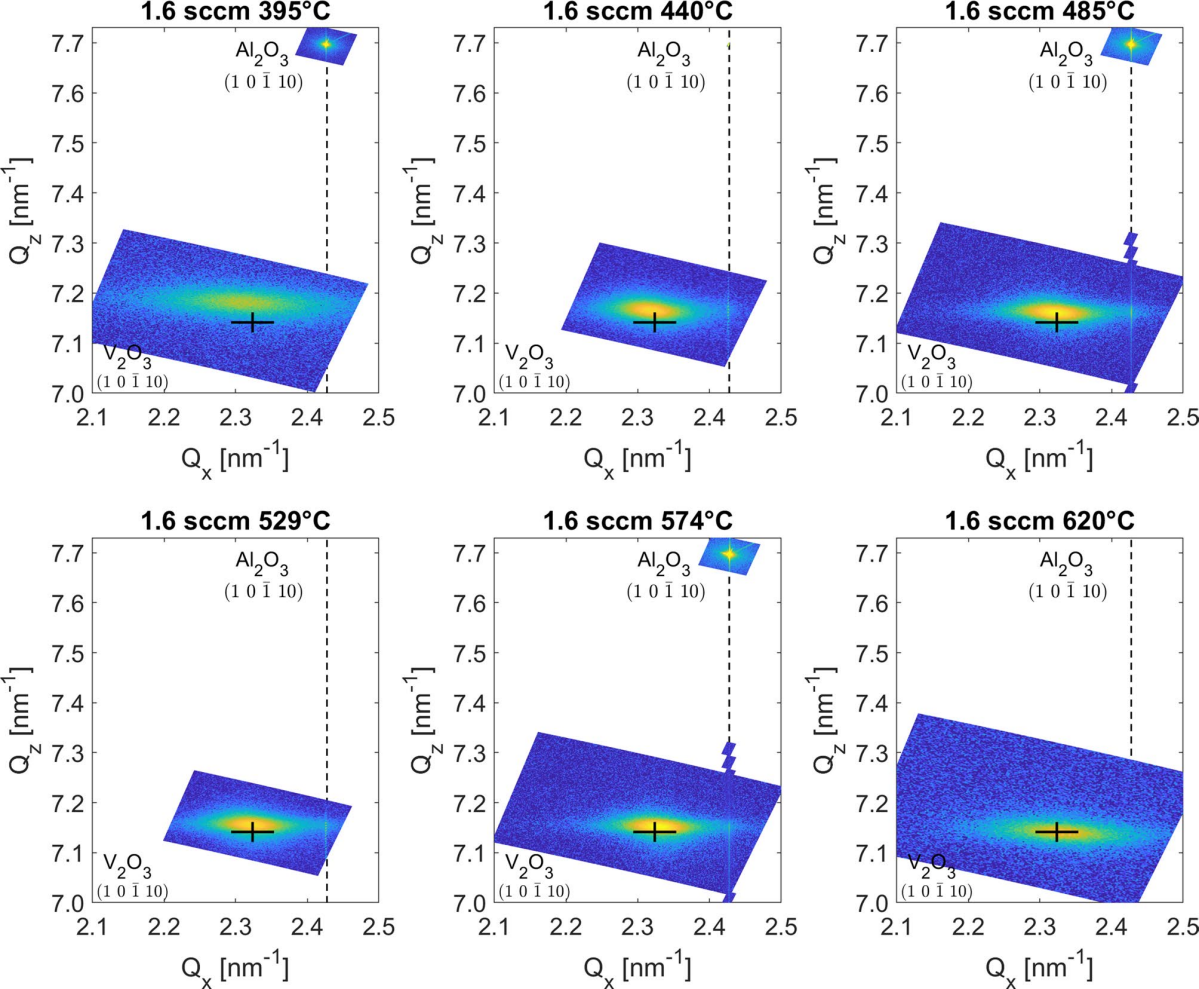
Overview of reciprocal space maps for the $\hbox {V}_{2}\hbox {O}_{3}$ film series grown at different substrate temperatures. The graphs show scans around the the (1 0 − 1 10) peak location of $\hbox {V}_{2}\hbox {O}_{3}$ as well as the same peak for the $\hbox {Al}_{2}\hbox {O}_{3}$ substrate, recorded for calibration of the X-ray instrument. The black cross marks the bulk value for the $\hbox {V}_{2}\hbox {O}_{3}$ (1 0 − 1 10) peak.

In order to investigate the crystallographic parameters of the films and the role of the deposition temperature, selected samples were scanned by reciprocal space maps. The RSM scans were focused on the (1 0 − 1 10) peak of the $\hbox {V}_{2}\hbox {O}_{3}$ film. This peak was chosen as it is the highest intensity asymmetrical peak of $\hbox {V}_{2}\hbox {O}_{3}$. Recording the X-ray intensity at an asymmetrical peak allows the determination of both the in-plane and out-of-plane lattice constants, *a* and *c* as well as a determination of the lateral correlation length and mosaicity of the film from the peak structure.

**Figure 2:**
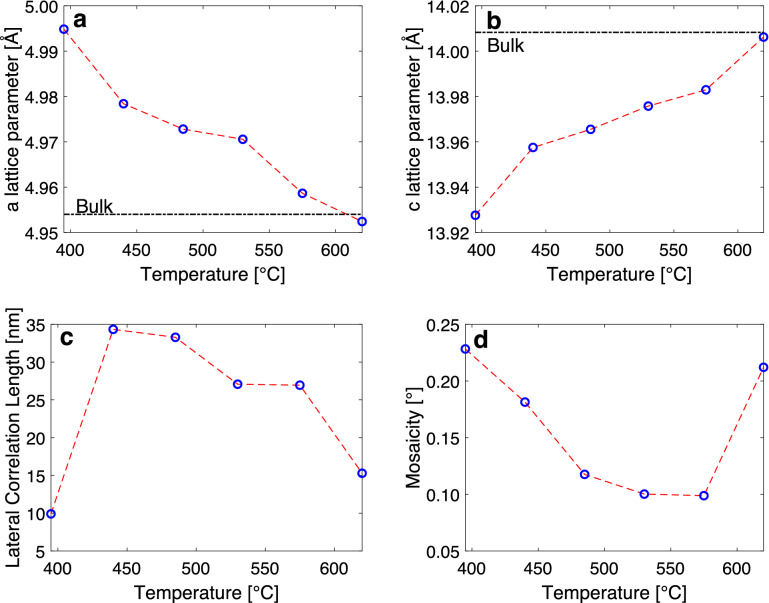
(**a**,**b**) The *a* and *c* lattice parameters and (**c**,**d**) the lateral correlation length and mosaicity extracted from the RSM of the (1 0 − 1 10) peak of $\hbox {V}_{2}\hbox {O}_{3}$ as a function of growth temperature.

Figure 1 shows RSM scans for a series of films grown at different deposition temperatures. The scans reveal peaks corresponding to relaxed $\hbox {V}_{2}\hbox {O}_{3}$ as well as a fully strained layer at the substrate interface with a lateral reciprocal space vector value corresponding to that of the underlying $\hbox {Al}_{2}\hbox {O}_{3}$ substrate. The values of the lattice parameters are strongly correlated to the deposition temperature. With increasing deposition temperature both the *a* and *c* lattice parameters relax reaching almost bulk values for the 620 $^{\circ }$C film. Figures 2a,b show that the relative change in the *a* lattice parameter is larger than in the *c* lattice parameter. In Fig. 2c,d, the lateral correlation length can be seen to increase within the temperature range 440–575 $^{\circ }$C, as well as the mosaicity decreasing. This change in the crystallographic parameters indicates that larger and better ordered crystals are obtained in that temperature range. However, it should be noted, that as the crystals increase in size, they might have internal stresses in them which will affect the MIT as proposed by Schuler et al.^10^.

#### Surface morphology

**Figure 3:**
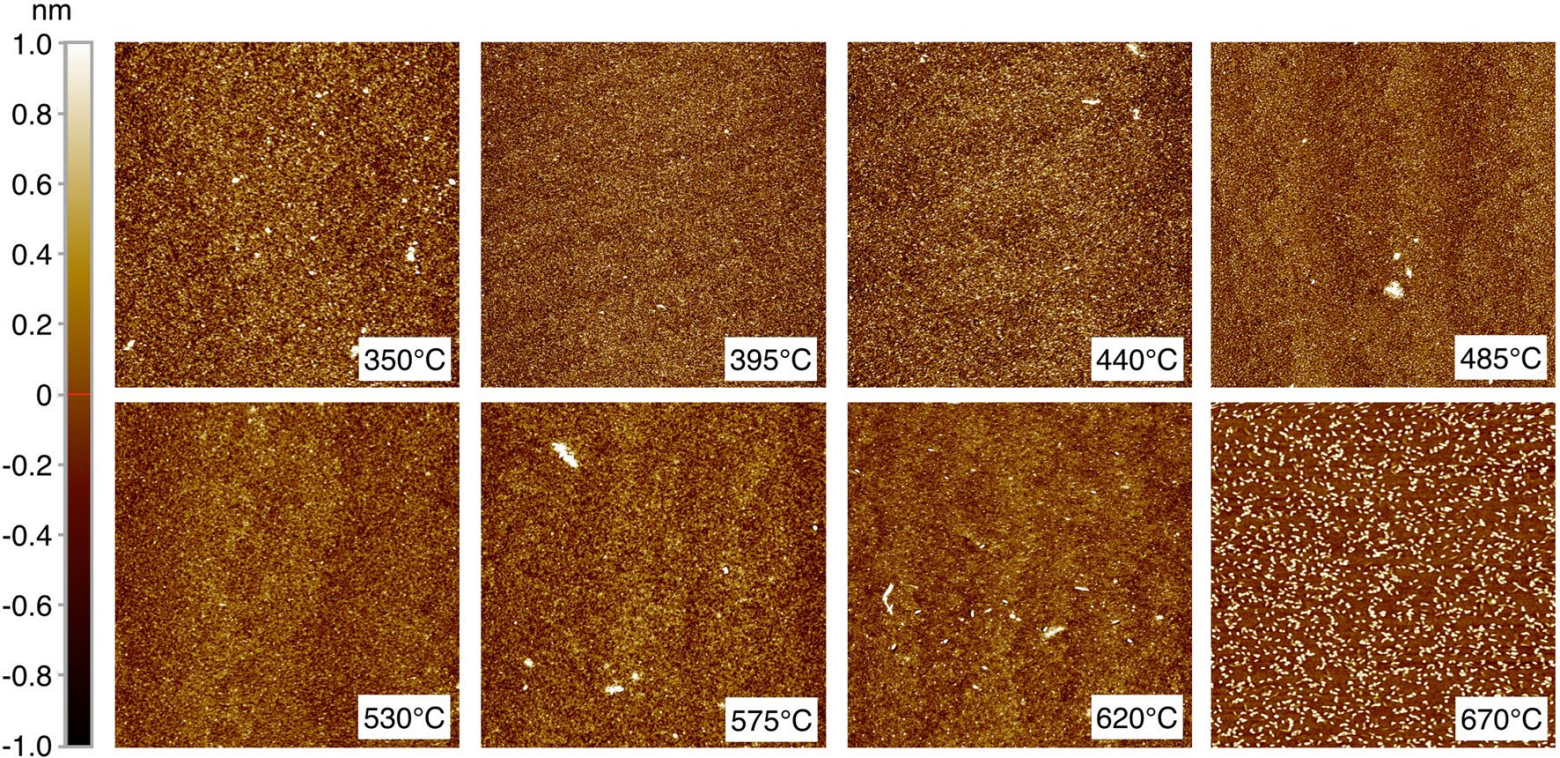
5 $\upmu$m $\times$ 5 $\upmu$m AFM images of the temperature series. Note, the color bar on left corresponds to all images except for 670 $^{\circ }$C, where the scale is from ± 4 nm instead of ± 1 nm. An overview of the RMS roughness value determined from the AFM scans is given in Fig. 4.

**Figure 4:**
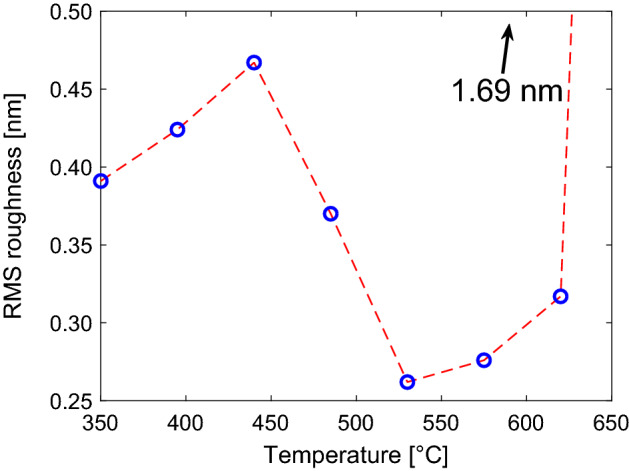
The root mean square roughness taken from the AFM images as a function of temperature (Fig. 3). The value for the 670 $^{\circ }$C film is cut off from the graph as it was an extreme outlier, its value was 1.69 nm.

Atomic force microscopy scans recorded for films deposited within the high quality epitaxy temperature window display low roughness surfaces exhibiting atomic terracing, see Fig. 3. Outside of this window the films exhibit a granular structure with increasing roughness. For the film grown at 670 $^{\circ }$C there is a clear change in morphology and crystal islands of 4–6 nm height can be seen. A similarly changed morphology with lesser extent is observed to form at 620 $^{\circ }$C. In Fig. 4, the root mean square roughness extracted from the images can be seen. Most of the films have similar roughness values of well below 0.5 nm. The films grown at 530 $^{\circ }$C and 575 $^{\circ }$C show considerably lower values approaching 0.25 nm. The surface roughness increases dramatically at 670 $^{\circ }$C where it is almost 4 times larger at 1.69 nm.

#### Electrical characterization

**Figure 5:**
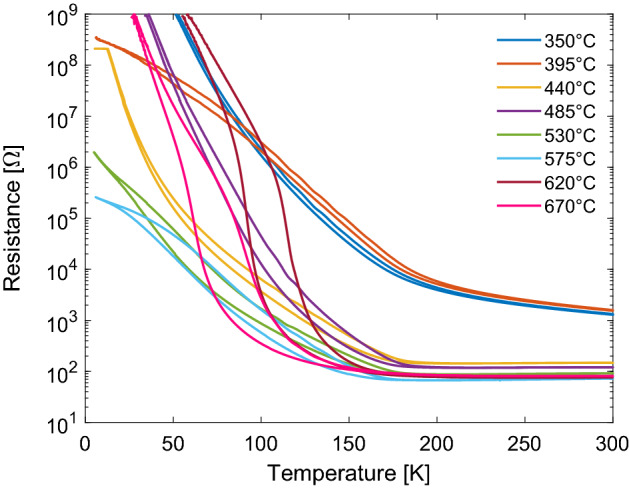
Resistance measurements as a function of temperature for films deposited under different substrate temperatures. The results reveal three distinct classes of resistance curves which can be classified into three types of films depending on their growth temperature. Type I ($<400\,^{\circ }$C) display a high room temperature resistance with a narrow hysteresis, type II films (grown at 400–600 $^{\circ }$C) display a lower room temperature resistance and a stronger hysteresis, and type III ($>600\,^{\circ }$C) show a sharper transition with a stronger hysteresis shifted to lower temperatures.

As the structural phase transition and MIT in $\hbox {V}_{2}\hbox {O}_{3}$ are linked, the easiest way of observing it is with resistance measurements. Figure 5 shows resistance measurements for both decreasing and increasing temperature between 10 and 300 K. The data was recorded for films fabricated at different deposition temperatures. Visible in the figure is the hysteresis associated with the first order phase transition of $\hbox {V}_{2}\hbox {O}_{3}$. For the films grown below 400 $^{\circ }$C, the films exhibit high resistance at room temperature with a very narrow hysteresis (type I). As the growth temperature is raised, the room temperature resistance is reduced and the hysteresis increases (type II). For temperatures above 600 $^{\circ }$C the transition becomes much sharper and a large hysteresis is seen (type III). However, the transition temperature is shifted to a lower value compared to the bulk value of around 155 K. This shift in transition temperature has been linked to the stoichiometry of the film^8^ as well as the local strain in the films^10^. The ratio between the vertical and lateral lattice parameters has been discussed as a governing factor in modifying the transition temperature^5^ both for overall thin film properties as well as under local stress induced using contact probe pressure^29^.

### Sputtering power dependence

The properties of $\hbox {V}_{2}\hbox {O}_{3}$ films are strongly dependent on the stoichiometry. Within this study several growth parameters are varied which affect directly not only the crystallographic structure and quality of the films but also the stoichiometry. The most apparent parameter affecting the stoichiometry is that of the amount of oxygen present in the chamber enabling the oxidation of the vanadium atoms sputtered from the vanadium target. The stoichiometry is therefore also dependent directly on the amount of vanadium atoms emanating from the vanadium target which can be controlled directly by the magnetron sputtering power.

#### XRD

**Figure 6:**
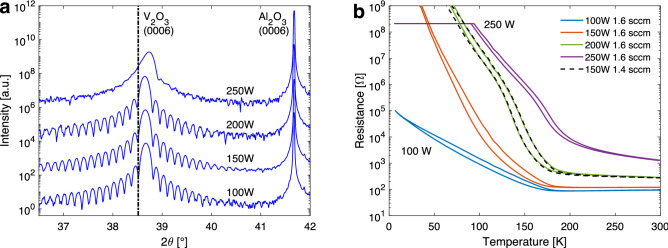
(**a**) X-ray diffraction scans and (**b**) resistance as a function of temperature for $\sim$60 nm thick $\hbox {V}_{2}\hbox {O}_{3}$ films deposited at different power settings with fixed $\hbox {O}_2$ flow of 1.6 sccm and deposition temperature of 485 $^{\circ }$C. The dashed line in (**a**) shows the angular peak position of bulk $\hbox {V}_{2}\hbox {O}_{3}$^30^. As the sputtering power is reduced the oxygen content of the $\hbox {V}_{2}\hbox {O}_{3}$ becomes higher increasing the amount of oxygen interstitials in the film suppressing the MIT and shifting it to lower temperatures. The dashed line shows the resistance recorded for a sample grown with a lower oxygen flow setting of 1.4 sccm at a sputtering power of 150 W. Although the growth parameters are different the curve corresponds closely to the film grown at a lower $\hbox {O}_2$ flow setting and higher power. Results presented in (**b**) are reproduced with permission from samples and data presented in supplementary Fig. S6 from Ref.^11^.

Figure 6a shows X-ray diffraction scans for a series of $\hbox {V}_{2}\hbox {O}_{3}$ films grown with different power settings while maintaining other parameters fixed. For this series the substrate temperature was 485 $^{\circ }$C and $\hbox {O}_2$ flow rate was 1.6 sccm. Thickness characterization by X-ray reflectivity revealed the growth rate to increase with the power setting with values of 55, 86, 121 and 163 pm/s for the 100, 150, 200 and 250 W settings, respectively. In order to maintain a fixed film thickness for the series the deposition time was controlled to compensate for the changing growth rate. For power settings in the range 100–200 W the films display a highly epitaxial nature with a strong $\hbox {V}_{2}\hbox {O}_{3}$ (0006) peak and Laue fringes extending on both sides revealing the vertical coherence length to be close to that of the film thickness. The peak position varies only slightly for the three films indicating that the general crystallographic nature is not changed substantially within this confined power range although it extends over a factor of two. At the highest tested setting of 250 W the crystal quality is reduced, although this film is still highly textured, with a corresponding decrease in peak intensity. This film furthermore displays a higher peak position indicating an increased compressive strain in the out-of-plane direction compared to the epitaxial films.

#### Electrical characterization

As has been shown in this paper, the deposition settings strongly affect the structural as well as electrical properties of $\hbox {V}_{2}\hbox {O}_{3}$ films. This is especially clear for the temperature dependence of the electrical resistance of the films as slight changes in deposition conditions, although not causing large changes in the structural quality of the films, can affect strongly the scale and magnitude of the MIT of the material^8,11^. Figure 6b shows the resistance of $\hbox {V}_{2}\hbox {O}_{3}$ films deposited at different magnetron sputtering power and $\hbox {O}_2$ settings. Similar to changes in the resistivity for films deposited at different $\hbox {O}_2$ flow settings the temperature dependent resistivity of the films is directly dependent on the sputtering power.

Comparing the results of resistance measurements for films deposited at higher power to films deposited at lower power but with a smaller $\hbox {O}_2$ flow setting reveals clear similarities. A higher power in a fixed $\hbox {O}_2$ flow setting effectively increases the metallic portion of sputtered flux from the magnetron increasing the V/O ratio in the films. Even though differences in the plasma chemistry are expected during growth with different power settings, such as for the ionization level and stoichiometry, the films show the same structural and transport properties. The metal–insulator transition can therefore be both directly tuned through the $\hbox {O}_2$ flow during deposition as well as through the power setting while maintaining a highly defined single crystalline structure. The control of the $\hbox {O}_2$ flow or sputtering power allows us to tune exactly the amount of oxygen interstitials (or excess oxygen in the film) at a given deposition temperature resulting in films with exactly the same transition behaviour. Any other means which can cause the same phenomenon (i.e. control in oxygen incorporation) can result in the same transition performance.

### Discussion

In this study we focus our attention on the effect of the growth settings on the structural and electrical properties of $\hbox {V}_{2}\hbox {O}_{3}$ films grown by reactive dc-magnetron sputtering. The electrical properties of the films show a strong dependence on the deposition conditions varying in magnitude and transition temperature, even when structural variations are observed to be minor from X-ray diffraction measurements.

**Figure 7:**
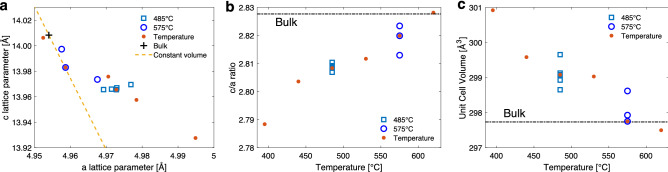
(**a**) The *c* lattice parameter plotted as a function of the *a* lattice parameters for all of the films. Constant unit cell volume is shown by the dashed line. Different growth series are labelled with different points. There are two oxygen flow rate series at fixed temperatures. At 485 $^{\circ }$C the flow rate ranges between 1.4 and 2.0 sccm, and at 575 $^{\circ }$C it ranges between 1.4 and 1.8 sccm. The temperature series was grown at the fixed oxygen flow rate of 1.6 sccm. (**b**) *c*/*a* ratio and (**c**) lattice unit cell volume as a function of growth temperature. The dashed lines correspond to bulk values for $\hbox {V}_{2}\hbox {O}_{3}$. Overlapping points correspond to the same data within different series.

Figure 7a shows the *c* lattice parameter as a function of the *a* lattice parameter for all of the films. It can be seen that there is a clear trend in the distribution of the lattice parameters. The unit cell expands preferentially with regards to the *a* lattice parameter at lower temperatures. From the results presented in the graph, the dominant factor in affecting the observed lattice parameters is the growth temperature while the oxygen flow rates affects the parameters to a lesser extent and preferentially the *a* lattice parameter. This result is further illustrated in Fig. 7b,c which shows the *c*/*a* ratio and unit cell volume as a function of growth temperature. Films grown at a higher temperature show a *c*/*a* corresponding closer to the bulk value with increasing growth temperature while films grown at lower temperatures reveal a lower *c*/*a* ratio. For the temperature series, films grown at the higher temperatures had larger *a* and smaller *c* lattice parameters than films grown at the lower temperatures with the highest temperature reaching bulk values in both the *a* and *c* lattice parameters. Although the point of origin for these values is shifted, this expansion of the *a* lattice parameter and compression of the *c* lattice parameter is in agreement with the thermal expansion of $\hbox {V}_{2}\hbox {O}_{3}$. With increasing temperature the *a* lattice parameter has been observed to increase while the *c* lattice parameter has been shown to decrease with increasing temperature up to $\sim$ 600 $^\circ$C^31^.

Figure 6b shows the resistance as a function of temperature for the series grown at 485 $^{\circ }$C with different sputtering power. The graph shows a clear change with an increase in the resistance and shift of the transition to higher temperatures with increasing power (i.e. with reduced oxygen content of the films). These results indicate that extra oxygen incorporated in the films sits at interstitial sites which give rise to an increase in the *a* lattice parameter while having much less impact on the *c* lattice parameter (Fig. 7a) and effectively a small change in the *c*/*a* ratio as seen in Fig. 7b. Comparatively larger changes are observed in the unit cell volume of the films as it scales proportionally more strongly with the in-plane lattice parameter. The increased strain in the *a* lattice parameter arising from the interstitial atoms leads to more stabilization of the metallic state, suppressing the formation of the insulating state^11^. These changes in the transition are in agreement with recently published results which show that interstitial oxygen defects as parts of O Frenkel pairs in $\hbox {V}_{2}\hbox {O}_{3}$ lower the energy cost of the transition and thereby shift the energy balance in the crystal towards the high temperature metallic phase, reducing the temperature of the transition^20^.

A similar series where the oxygen flow rate was varied but at a higher growth temperature of 575 $^{\circ }$C revealed an increased *a* lattice parameter with the oxygen flow setting but substantially smaller *c* lattice parameter compared to the 485 $^{\circ }$C series in accordance with the *c* lattice parameter being closer to bulk values. With the higher growth temperature the films relax towards bulk values of the *c* and *a* lattice parameters (Fig. 2a,b) with a larger relative change in the *a* lattice parameter and larger impact on the *c*/*a* ratio. Coupled to this reduction in the *a* value the probability of inhabiting oxygen interstitials is reduced and the suppression of the transition is lessened as can be seen in Fig. 5 where films grown at a higher temperature reveal a more pronounced MIT.

Changing the deposition temperature we observe three distinct types of transitions. Films grown at low temperatures, below 400 $^{\circ }$C display a high room temperature resistance with a narrow hysteresis shifted to higher temperatures (type I). Films grown in the temperature range 400– 600 $^{\circ }$C display a stronger hysteresis and lower room temperature resistance (type II). At the higher temperatures (above 600 $^{\circ }$C) the films display a sharper transition with a larger hysteresis which is shifted to lower temperatures (type III). The higher room temperature resistance of the type I could be due to less crystallinity of the films as can be seen in the reciprocal space maps shown in Fig. 1 which display broader peaks of lower intensity compared to the other films. Type I films should therefore include more domain boundaries which act as scattering points for electron conduction. For type II films the crystallinity of the films is well defined. These films display transitions which can be directly tuned through the growth conditions i.e. their electronic properties are directly dependent on the stoichiometry via oxygen interstitials. In type III, the films are fully relaxed and act similar to bulk $\hbox {V}_{2}\hbox {O}_{3}$ thus showing wider hystereses and more prominent transitions although they are shifted towards lower temperatures as the growth temperature is increased.

**Figure 8:**
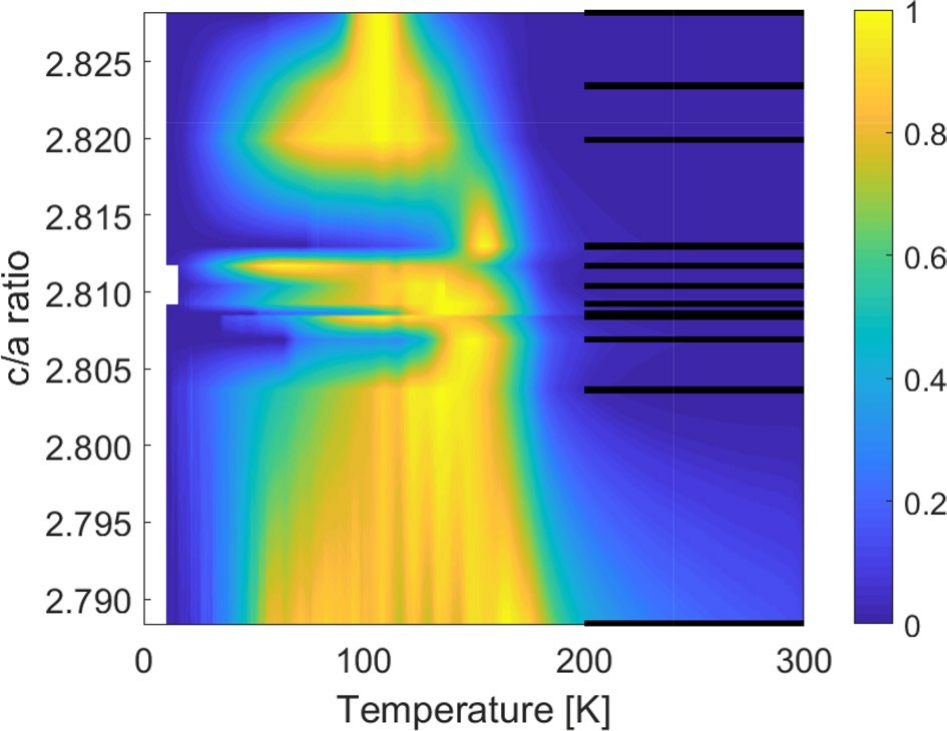
Combined plot revealing the temperature of the transition from all the samples investigated in the study highlighting the hysteresis in the resistance of the films with temperature. The colourscale shows the normalized difference of the logarithm of the resistance, ($\log _{10}(R_{\mathrm {heating}}) - \log _{10}(R_{\mathrm {cooling}})$) divided by the maximum difference for each measurement, as a function of temperature. The black lines show the determined *c*/*a* values for the samples.

Previous results published in the literature have observed a correlation between the temperature and magnitude of the MIT and the *c*/*a* ratio of the lattice parameters^5,32,33^. Figure 8 shows a combined plot of the electrical resistance of the films fabricated for this study arranged according to their *c*/*a* values. The graph highlights the onset temperature of the MIT and the extent of the hysteresis in the transition with respect to the *c*/*a* values. The onset temperature of the transition shows a clear dependence on the *c*/*a* ratio increasing in temperature with decreasing *c*/*a* value. The extent of the hysteresis shows, however, limited dependence on the *c*/*a* value. This results is especially clear for films with transitions of type II which have a *c*/*a* ratio in the range 2.805–2.815 where the amount of oxygen interstitials affects the transition strongly. These results therefore clearly illustrate that the crystallographic lattice parameters are not necessarily the dominant factors in controlling the MIT of reactively sputtered $\hbox {V}_{2}\hbox {O}_{3}$ thin films even though structural analysis reveals the films to be of a highly crystalline nature. Changes in the MIT can therefore not be directly linked to epitaxial strain in the films as the role of defects, interstitial oxygen and local strain in the film can not be neglected when investigating the coupling between the crystallographic nature of the films and the MIT.

## Conclusions

We have shown that using reactive magnetron sputtering, highly epitaxial thin films of $\hbox {V}_{2}\hbox {O}_{3}$ can be grown on sapphire substrates. The high level of crystallinity can be reached at temperatures substantially lower than reported previously within a 200 $^{\circ }$C wide temperature range. Within this range the surface roughness of the films is below 1 nm and the films reveal atomically flat terraced structures. We observe the growth temperature to have a direct effect on the nature, scale and magnitude of the MIT. Films grown at temperatures below 400 $^{\circ }$C (type I) display a narrow hysteresis shifted to higher temperatures and resistance values. Films grown within the temperature window 400–600 $^{\circ }$C display a stronger hysteresis (type II) which can be tuned by the sputtering power and $\hbox {O}_2$ flow settings. Films grown at elevated temperatures (type III) are fully relaxed and display more prominent transitions at reduced temperatures compared to bulk $\hbox {V}_{2}\hbox {O}_{3}$. Comparing films of type II grown under different growth conditions, we observe that altering the sputtering power with fixed oxygen flow rates affects the properties in a similar way as for controlling the oxygen conditions in the chamber with both cases moderating the amount of oxygen interstitials in the films. With decreasing deposition temperature we observe a preferential increase in the *a* lattice parameter with a concomitant decrease in the *c* lattice parameter and increase in the unit cell volume. A clear dependence of the onset temperature of the MIT is observed with the *c*/*a* ratio while the extent of the transition hysteresis is observed to be independent from the *c*/*a* ratio. A clear correlation of the MIT with the fabrication conditions is however observed indicating that small scale variations in the stoichiometry, defects and local strain in the film can have a controlling effect on the properties of the MIT.

## Methods

### Thin film growth

The $\hbox {V}_{2}\hbox {O}_{3}$ thin films studied in this work were all fabricated by reactive dc-sputtering using a custom built magnetron sputtering chamber^34^. A vanadium target with 99.5% purity was used and the sputtering power, substrate temperature and $\hbox {O}_2$ flow setting controlled and maintained fixed for each film deposition. Prior to deposition the base pressure of the system was below $4\times 10^{-6}$ Pa. The sputtering gas was 99.999% pure argon gas with a fixed flow setting of $q_{\mathrm Ar} = 20$ sccm throughout the deposition. Oxygen gas of purity 99.999% was used at flow rate settings ranging from $q_{\mathrm {O}_2} =1.4$ sccm up to 2.0 sccm. The chamber pressure during sputtering was maintained at 0.4 Pa using a throttle valve in front of the turbomolecular pump. For this study we use $1\times 1$ cm$^2$ single crystalline sapphire substrates with *c*-plane [0001] surface orientation.

During growth the substrate temperature was controlled in the range of 300–700 $^{\circ }$C using a 3.8 cm diameter circular plate heater located 4 mm below the substrate holder. The deposition temperature corresponds to the temperature on the sample holder as determined by calibration of the temperature as a function of the heater power settings. The sputtering power was varied between 100 and 250 W. Prior to insertion into the vacuum chamber, the substrates were cleaned, sequentially, in ultrasound with acetone, methanol and isopropanol, for 5 min each. Following the chemical cleaning, the samples were rinsed with deionized water, dried with $\hbox {N}_2$ and subsequently put into the load lock of the vacuum chamber. Under vacuum the substrates were annealed at 620 $^{\circ }$C for 20 minutes and then allowed to reach their respective deposition temperature for 15 min. Before deposition the target was pre-sputtered in pure argon for 8 min, followed by another 7 min in the intended argon/oxygen mixture before opening the shutter.

The chamber pressure was monitored with 2 different gauges. Firstly, a full range combined pirani and cold cathode gauge, for high and low pressure measurement, capable of measuring from atmosphere down to $5\times 10^{-7}$ Pa. Secondly, a capacitance manometer, for high accuracy measurement of the absolute pressure during growth, operating in the range between 10 Pa down to 0.001 Pa.

The thickness of the films was maintained at $\sim \, 60$ nm through timing of a shutter located in front of the sputtering magnetron. After finishing the deposition, the power to the heater was turned off and the films allowed to cool to room temperature before being retrieved for ex-situ characterization.

#### Structural characterization

The structural properties of the films, which comprises the focus of this study, were investigated by X-ray diffraction (XRD), X-ray reflectivity (XRR) and reciprocal space mapping (RSM) measurements using a Panalytical X’Pert Pro diffractometer at room temperature.

For the X-ray measurements there were 2 different optic setups. Firstly for the 2$\theta$/$\omega$ scans a 2 bounce hybrid monochromator with 1/8$^{\circ }$ slit was used on the incident side, while the diffracted side had a parallel plate collimator (0.27$^{\circ }$) with a 0.1 mm slit. Secondly, for the $\omega$ rocking curves and RSM’s, the incident side had the hybrid monochromator while on the diffracted side there was a triple axis analyzer crystal with a 12 $^{\prime \prime }$ acceptance angle.

From the XRR measurements the thickness and density of the films were determined by fitting using the X’Pert Reflectivity program. For all the films the density was close to bulk value, being in the range 4.95–5.02 g/cm$^{3}$, whereas the bulk value is 5.02 g/cm$^{3}$^30^. The thickness of the films investigated in this study was between 55 and 65 nm. X-ray diffraction measurement and reciprocal space mapping were utilized to determine the internal crystallographic parameters, such as the in-plane and out-of-plane lattice parameters, lateral correlation length and mosaicity of the films.

The surface morphology of the films was investigated by atomic force microscopy using a Park XE-100 instrument in both contact as well as non-contact mode.

#### Electrical characterization

The resistance of the films was recorded using a mini cryogen free system from Cryogenic Uk ltd. Measurements were performed with a 2 point setup using a Keithley 2400 sourcemeter connected to a Keithley 7001 switch system with a 7012-S 4 $\times$ 10 matrix card. With this setup it was possible to measure 2 samples simultaneously. The sourcemeter and the switch card have 10 G$\Omega$ and 1 G$\Omega$ input resistances respectively limiting the maximum resistance measurable to approximately 5 G$\Omega$.

To connect leads to the sample, contacts were e-beam evaporated onto the edges of the films using a shadow mask. These contacts consisted of 5 nm of chromium followed by 100 nm of gold. Wires from the system were attached to the contacts using conductive silver paint. Each sample was scanned decreasing the temperature from 300 to 10 K followed by a second scan increasing the temperature from 10 K up to 300 K. Both temperature scans were performed at a scan rate of 0.8 K/min.
